# Nasal and Fecal Microbiota and Immunoprofiling of Infants With and Without RSV Bronchiolitis

**DOI:** 10.3389/fmicb.2021.667832

**Published:** 2021-06-01

**Authors:** Claudio Alba, Marina Aparicio, Felipe González-Martínez, María Isabel González-Sánchez, Jimena Pérez-Moreno, Blanca Toledo del Castillo, Juan Miguel Rodríguez, Rosa Rodríguez-Fernández, Leonides Fernández

**Affiliations:** ^1^Department of Nutrition and Food Science, Complutense University of Madrid, Madrid, Spain; ^2^Department of Pediatrics, Hospital Universitario Gregorio Marañón, Madrid, Spain; ^3^Instituto de Investigación Sanitaria Gregorio Marañón (IISGM), Madrid, Spain; ^4^Department of Galenic Pharmacy and Food Technology, Complutense University of Madrid, Madrid, Spain

**Keywords:** bronchiolitis, respiratory syncytial virus, immunoprofile, nasal lavage, *Haemophilus*, BAFF/TNFSF13B, bacterial microbiota

## Abstract

Bronchiolitis associated with the respiratory syncytial virus (RSV) is the leading cause of hospitalization among infants aged < 1 year. The main objective of this work was to assess the nasal and fecal microbiota and immune profiles in infants with RSV bronchiolitis, and to compare them with those of healthy infants. For this purpose, a total of 58 infants with RSV-positive bronchiolitis and 17 healthy infants (aged < 18 months) were recruited in this case-control study, which was approved by the Ethics Committee of the Hospital Gregorio Marañón. Nasal and fecal samples were obtained and submitted to bacterial microbiota analysis by 16S rDNA sequencing and to analysis of several immune factors related to inflammatory processes. Nasal samples in which *Haemophilus* and/or *Moraxella* accounted for > 20% of the total sequences were exclusively detected among infants of the bronchiolitis group. In this group, the relative abundances of *Staphylococcus* and *Corynebacterium* were significantly lower than in nasal samples from the control group while the opposite was observed for those of *Haemophilus* and *Mannheimia*. Fecal bacterial microbiota of infants with bronchiolitis was similar to that of healthy infants. Significant differences were obtained between bronchiolitis and control groups for both the frequency of detection and concentration of BAFF/TNFSF13B and sTNF.R1 in nasal samples. The concentration of BAFF/TNFSF13B was also significantly higher in fecal samples from the bronchiolitis group. In conclusion, signatures of RSV-associated bronchiolitis have been found in this study, including dominance of *Haemophilus* and a high concentration of BAFF/TNFSF13B, IL-8 and sTNF.R1 in nasal samples, and a high fecal concentration of BAFF/TNFSF13B.

## Introduction

Respiratory syncytial virus bronchiolitis (RSV) is the leading cause of hospitalization in infants during their first year of life and one of the most frequent causes of visits in pediatric emergencies. RSV produces a broad spectrum of diseases, ranging from a mild infection of the upper airway to a severe infection of the lower airway (Leader and Kohlhase, 2002). The factors that explain the different severity of this disease in each patient are diverse and include both host factors, such as the existence of underlying malformations, prematurity, bronchopulmonary dysplasia or congenital heart disease and viral factors, including viral load or viral genotypes (García et al., 2010; Rodriguez-Fernandez et al., 2017). Most likely the severity of the disease is influenced by a combination of both types of factors and, also, by a dysregulated or impaired host innate immune response (de Steenhuijsen Piters et al., 2016). In addition, it is believed that the composition of the respiratory and fecal microbiotas may modulate the host immune response in the bronchiolitis setting. In this context, the principal aim of this study was to assess the nasal and fecal bacterial microbiotas and immune profiles of infants with RSV bronchiolitis and to compare them with those of healthy ones.

## Materials and Methods

### Study Design and Participants

**FIGURE 1:**
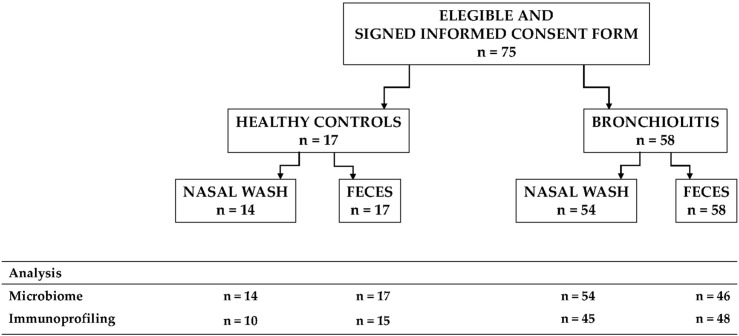
Flowchart of the participants included in the study, the samples collected and the analyses that were performed.

A case-control study to investigate the microbiota in nasal secretions and fecal samples from infants with RSV-positive bronchiolitis and from healthy infants was conducted. Infants > 24 months of age, with a previous episode of wheezing or respiratory distress, immunodeficiency, chronic underlying diseases, or treatment with immunosuppressive agents were excluded. We recruited 58 infants with RSV-positive bronchiolitis from the Department of Pediatrics of the Hospital Universitario Gregorio Marañón (Madrid, Spain) over a 17 months period from January 2017 to May 2018 (Figure 1). Parallel, we also included healthy infants (*n* = 17) without allergy, infection or respiratory symptoms. Sample size was estimated according to previous results describing four different microbiota profiles in infants hospitalized with bronchiolitis and the smallest difference for the bacterial abundance of the most representative genera recorded between two profiles (Hasegawa et al., 2016a).

Bronchiolitis was defined as the presence of cough, rhinitis, wheezing, tachypnea, crackles, use of accessory muscles, and/or nasal flaring with or without fever according to the American Academy of Pediatrics (American Academy of Pediatrics Subcommittee on Diagnosis and Management of Bronchiolitis., 2006). Recurrent wheezing was defined by the presence of three or more episodes of physician-diagnosed wheezing within the 12 months after enrollment. RSV diagnosis was established using a direct immunofluorescence antibody (DFA) assay per standard of care and the results were confirmed by PCR in nasal wash samples obtained at enrollment (Bhandary and Boloor, 2016; Do et al., 2012). Informed consent from the parents or legal representatives of all participants was obtained. The study was approved by the Ethics Committee of the Hospital Gregorio Marañón (CEIC 276/16).

### Data and Sample Collection

Clinicians recorded demographic, medical, and home environment data using a clinical questionnaire at enrollment, and by chart review. Clinical disease severity parameters were assessed using a standardized severity score at admission (González Caballero and González Pérez-Yarza, 2001), as well as need for supplemental oxygen, pediatric intensive care unit (PICU), and length of hospitalization. The most relevant characteristics of the participants are summarized in Table 1.

**TABLE 1:** Participant characteristics.

Characteristic	Healthy	Bronchiolitis	*p* value*
Number of participants	17	58	
**Gender**			
Female	10 (59)	32 (55)	0.791
Male	7 (41)	26 (45)	
**Age**			
<4 months	12 (71)	29 (50)	0.134
≥4 months	5 (29)	29 (50)	
**Born preterm**			
No	15 (88)	54 (93)	0.613
Yes	2 (12)	4 (7)	
**Delivery**			
Vaginal	11 (65)	45 (78)	0.345
Caesarean section	6 (35)	13 (22)	
**Breastfeeding**			
No	4 (24)	14 (24)	1.000
Yes	13 (76)	44 (76)	
**Nutritional status**			
Normal	13 (76)	53 (91)	0.196
Malnourished	4 (24)	5 (9)	
Weight (percentile)	30.0 (22.3–37.7)	36.8 (32.2–41.4)	0.120
**Attending day care center**			
No	17 (100)	46 (79)	0.104
Yes	0 (0)	10 (17)	
Unknown	0 (0)	2 (3)	
**Tobacco exposure**			
No	12 (71)	38 (66)	**0.009**
Yes	1 (6)	18 (31)	
Unknown	4 (24)	2 (3)	

Values are number (percentage) of participants, except mean (95% confidence interval) for weight. *Fisher exact tests were used to evaluate differences in frequencies of the analyzed parameters except Chi-squared tests for gender and age (unknowns were not taken into consideration) and Wilcoxon rank sum test to evaluate differences in weight (percentile). Bold font indicates values having differences that are statistically significant.

One nasal wash was obtained from each patient, using a standardized protocol (Stewart et al., 2017), within 24 h of hospitalization (cases) or during the routine visit (controls). Parallel, one fecal sample from each infant was collected directly from the diaper in the first 2 days of admission (cases) or during the routine visit (controls). All the collected samples were immediately stored at −20 °C.

### Metataxonomic Analysis of Bacterial Microbiota

Nasal samples (1 g) from each infant were used for DNA extraction, which was performed as described (Pérez et al., 2019). Parallel, DNA was extracted from fecal samples (1 g) as already described (Lackey et al., 2019). 16S rRNA gene amplification and sequencing, targeting the V3-V4 hypervariable regions of the 16S rRNA gene, was performed in the MiSeq system of Illumina at the facilities of Parque Científico de Madrid (Tres Cantos, Spain) (Aparicio et al., 2020; Klindworth et al., 2013). The pooled, purified and barcoded DNA amplicons were sequenced using the Illumina MiSeq pair-end protocol (Illumina Inc., San Diego, CA, United States).

Taxonomical analyses of the V3-V4 region of the 16S rRNA amplicon data were conducted using MiSeq Reporter analysis software. Bioinformatic analysis was conducted combining the R version 3.5.1 (R Core Team, 2013)^1^, QIIME pipelines (v 1.9.1) (Caporaso et al., 2010) and Calypso (v 8.84) (Zakrzewski et al., 2017). A table of OTU counts per sample was generated, and bacterial taxa abundances were normalized to the total number of sequences in each sample. Sequences were rarified to 100,000 sequences for statistical analyses. Alpha diversity was assessed using the Shannon and Simpson diversity indices (Haegeman et al., 2013). Beta diversity studies were performed using the Principal Coordinates Analysis (PCoA) to plot patterns of bacterial community diversity through a distance matrix containing a dissimilarity value for each pairwise sample comparison. Quantitative (relative abundance) and qualitative (presence/absence) analyses were performed with the Bray-Curtis and binary Jaccard indices, respectively. PERMANOVA analysis with 999 permutations were used to reveal statistically significant differences.

### Immunoprofiling

Nasal samples (1 mL) were centrifuged and the supernatants were used for the immunological assays. Fecal samples were prepared as described previously (Aparicio et al., 2020). The concentrations of a wide array of inflammation-related immune factors (APRIL/TNFSF13, BAFF/TNFSF13B, Chitinase 3-like 1, IFNα, IFNβ, IFNγ, IL2, IL8, IL10, IL11, IL12p40, IL12p70, IL19, IL20, IL22, IL26, IL27p28, IL28/IFNλ2, IL29/IFNλ1, IL32, IL34, IL35, LIGHT/TNFSF14, MMP-1, MMP-2, MMP-3, osteocalcin, osteopontin, pentraxin 3, TSLP, TWEAK/TNFSF12, gp130/sIL-6Rb, sCD30/TNFRSF8, sCD163, sIL-6Ra, sTNF-R1, and sTNF-R2) were determined using the Bio-Plex Pro Human Inflammation Assay kit (Bio-Rad, Hercules, CA, United States) in the Bio-Plex 200 instrument (Bio-Rad). Every assay was run in duplicate and standard curves were performed for each analyte.

### Statistical Analysis

Quantitative data were expressed as the median and interquartile range (IQR). Differences between control and bronchiolitis groups were assessed using Fisher exact (or Chi-squared) tests for categorical variables and Wilcoxon rank sum tests with Bonferroni adjustment for multiple comparisons for quantitative variables. Principal Components Analysis (PCA) was performed to examine for similarities among nasal samples from bronchiolitis or control groups, using FactoMineR package in R. The strength and direction of association between variables was measured using the Spearman’s rank-order correlation analyses and visualized using R package *corrplot*. Statistical analysis and plotting were performed in the R environment (version 3.5.1 with library *ggplot*2 (Wickham, 2016). Differences were considered statistically significant at *p* < 0.05.

## Results

### Infants With Bronchiolitis Display an Altered Nasal Bacterial Microbiota

Baseline characteristics of the enrolled infants did not differ, but a higher proportion of infants with bronchiolitis were exposed to tobacco compared to controls (OR = 5.68; 95% CI, 0.68–47.15; *p* = 0.069) (Table 1).

**FIGURE 2:**
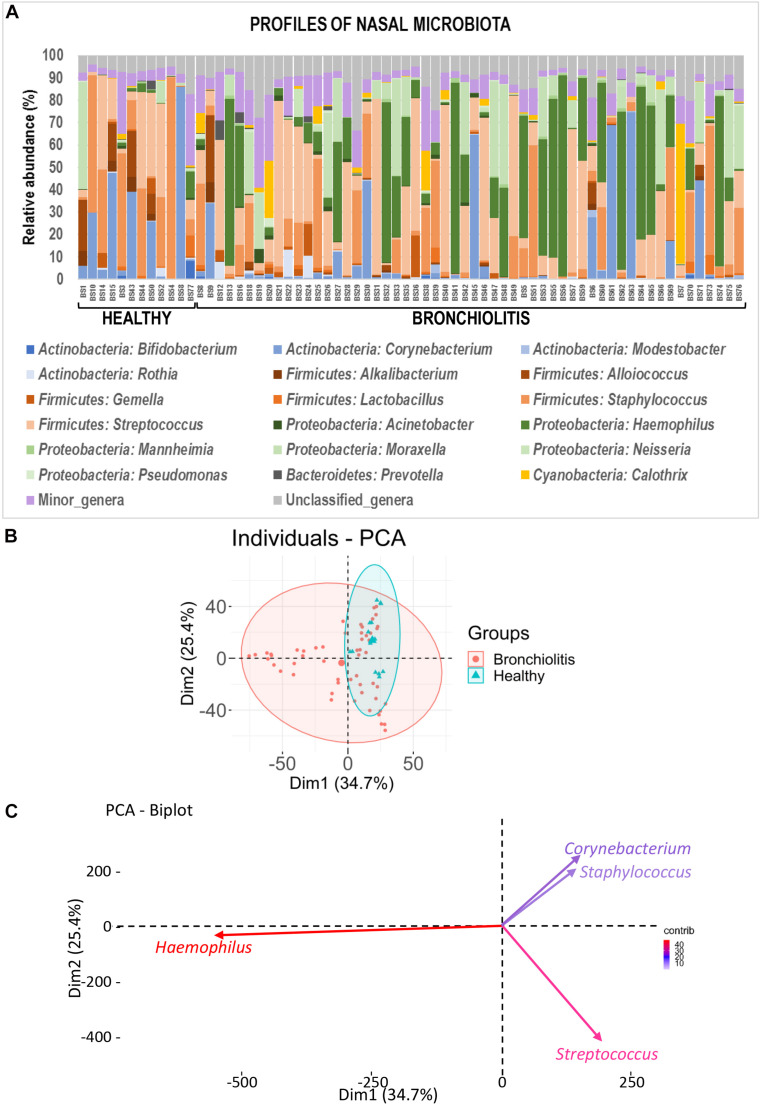
Nasal microbiota in healthy controls and bronchiolitis groups. **(A)** Individual profiles of the microbiota composition at the genus level (the 20 most abundant genera) of nasal samples from healthy control and bronchiolitis groups. Each stacked bar represents one sample and depicts the average relative abundance of the most abundant bacterial genera (color-coded). **(B)** Principal component analysis (PCA) for nasal samples based on the bacterial composition at the genus level showing the sample distribution around centroids (ellipses were defined by a 95% confidence interval). **(C)** Most influential genera (*cos*^2^ > 0.2) on sample separation presented in panel **(B)**.

**FIGURE 3:**
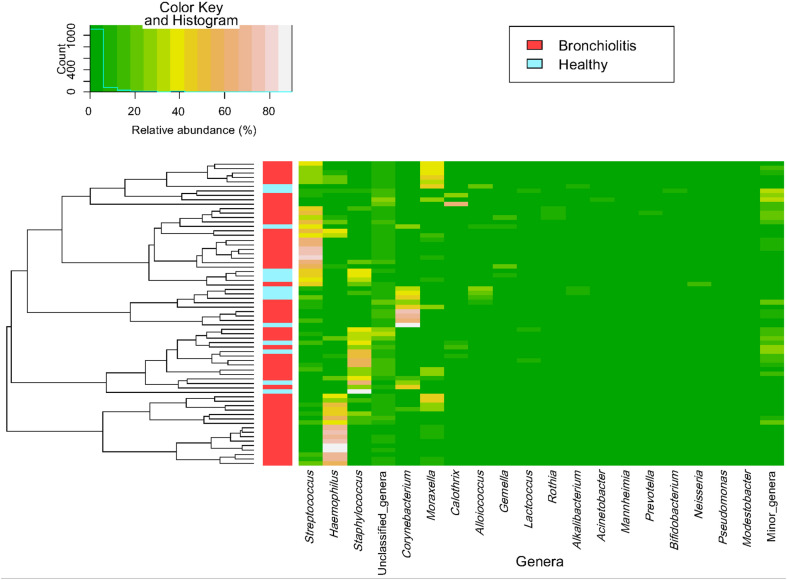
Heatmap of bacterial profile at the genus level in the microbiota of nasal samples. The plot depicts the relative abundance of each genera (*x*-axis clustering) within each sample (*y*-axis clustering). The relative abundances of the genera are represented by color intensity. The vertical bar (left) identifies samples from control (*n* = 14; blue color) and bronchiolitis (*n* = 54; red color) groups.

The individual bacterial microbiota patterns of nasal samples from control group were highly similar, while those from the bronchiolitis group were more diverse (Figure 2A). The main genera contributing to the distinctive grouping of the nasal samples according to their bacterial diversity were *Haemophilus*, *Streptococcus*, *Corynebacterium* and *Staphylococcus* (Figures 2B,C). The nasal samples clustered in five groups characterized by a high abundance of *Haemophilus* (24% of samples), *Staphylococcus* (22%), *Corynebacterium* (13%), *Streptococcus* combined or not with *Haemophilus* or *Staphylococcus* (26%), and *Moraxella*/*Streptococcus* (15%) (Figure 3). All clusters included samples from both control and bronchiolitis groups, except the *Haemophilus* cluster that only contained samples from the bronchiolitis group. In addition, all samples containing > 20% of *Haemophilus* and/or *Moraxella* sequences corresponded to the bronchiolitis group.

**FIGURE 4:**
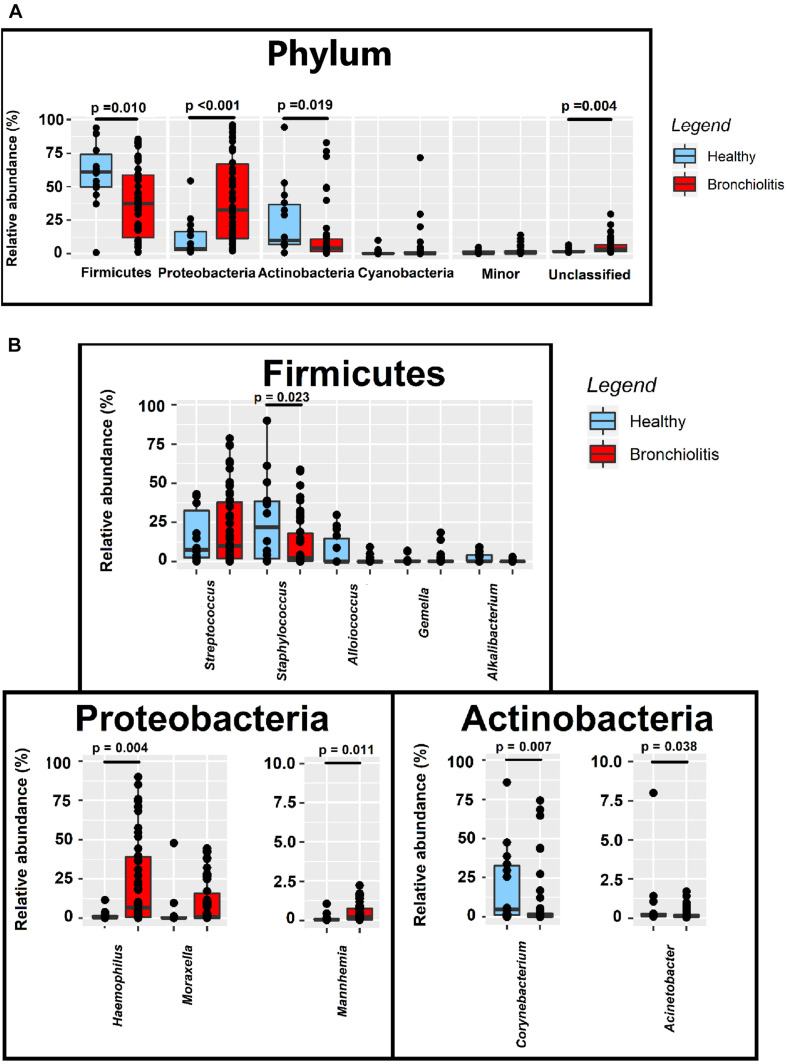
Relative abundance sequences at the phylum **(A)** and genus **(B)** level in nasal samples from healthy (*n* = 14; blue boxes) and bronchiolitis (*n* = 54; red boxes) infants. Significant differences between samples from both groups of infants are indicated (Wilcoxon rank sum tests).

The relative abundances of Firmicutes and Actinobacteria in the nasal samples from controls (median [IQR] = 61.02% [49.84–74.03%] and 9.97% [6.98–36.62%], respectively) were nearly twice compared to those from infants with bronchiolitis (median [IQR] = 37.41% [12.09–58.69%] and 4.22% [1.16–10.96%], respectively; Wilcoxon rank sum tests, *p* = 0.010 and *p* = 0.019) (Figure 4 and Supplementary Table 1). In contrast, the relative abundance of Proteobacteria in nasal samples from infants with bronchiolitis was about 9 times higher than in the controls (median [IQR] = 32.64% [11.21–66.89%] and 3.72% [2.48–16.42%], respectively; Wilcoxon rank sum tests, *p* < 0.001) (Figure 4 and Supplementary Table 1). At the genus level, the relative abundances of *Staphylococcus* and *Corynebacterium* in samples from the bronchiolitis group were 10 times lower than in samples from the control group (Wilcoxon rank sum tests, *p* = 0.023 and *p* = 0.007, respectively), while those of *Haemophilus* and *Mannheimia* were 30- and 10-fold higher (Wilcoxon rank sum tests, *p* = 0.004 and *p* = 0.011, respectively) (Figure 4 and Supplementary Table 1).

**FIGURE 5:**
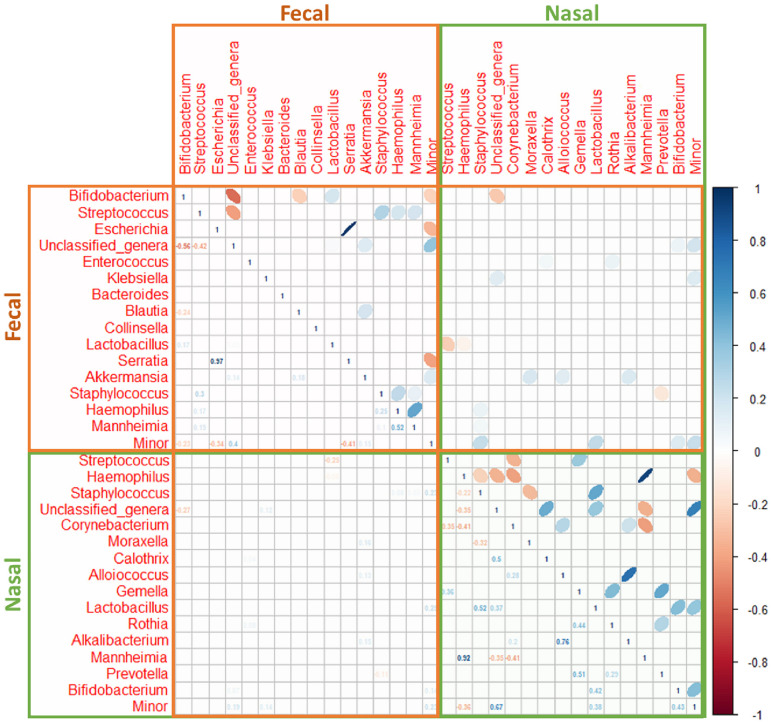
Spearman’s rank correlation analysis of the most abundant bacteria at the genus level in nasal (green box) and fecal (brown box) samples from both control (*n* = 9) and bronchiolitis (*n* = 34) groups. Blue ellipses represent positive correlations and red ellipses represent negative correlations. Only statistically significant correlations (*p* < 0.050) are shown).

A strong positive correlation between the relative abundance of the pairs *Haemophilus*/*Mannheimia* (ρ = 0.92) and *Alkalibacterium*/*Alloiococcus* (ρ = 0.76) and a weak negative correlation between *Haemophilus*/*Corynebacterium* (ρ = −0.41) and *Mannheimia*/*Corynebacterium* (ρ = −0.41) was recorded in nasal samples (Figure 5).

### Fecal Microbiota of Infants With Bronchiolitis Is Similar to That of Healthy Infants

**FIGURE 6:**
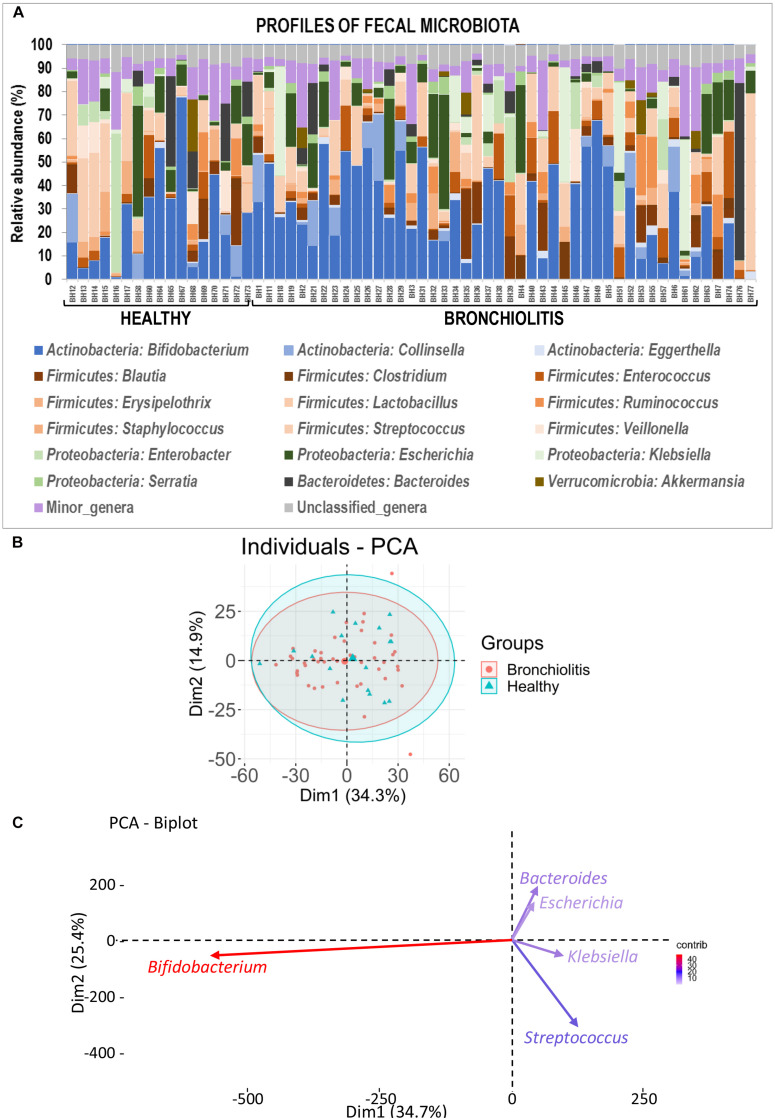
Fecal microbiota in healthy controls and bronchiolitis groups. **(A)** Individual profiles of the microbiota composition at the genus level (the 20 most abundant genera) of nasal samples from healthy control and bronchiolitis groups. Each stacked bar represents one sample and depicts the average relative abundance of the most abundant bacterial genera (color-coded). **(B)** Principal component analysis (PCA) for nasal samples based on the bacterial composition at the genus level showing the sample distribution around centroids (ellipses were defined by a 95% confidence interval). **(C)** Most influential genera (*cos*^2^ > 0.2) on sample separation presented in panel **(B)**.

Globally, the taxonomic profiles at the genus level of the individual fecal and nasal bacterial microbiotas were distinct (Figures 2, 6). The profiles of fecal microbiota were similar in the control and bronchiolitis groups regarding the most abundant genera (*Bifidobacterium*, *Streptococcus*, *Escherichia*) and changes were observed only on less abundant genera (Table 2). The relative abundance of *Staphylococcus* was higher in fecal samples from healthy infants than in those with bronchiolitis (median [IQR] = 0.30% [0.04–5.79%] and 0.05% [0.04–0.09%], respectively; Wilcoxon rank sum tests, *p* = 0.046). *Haemophilus* and *Eggerthella* were detected at a low rate in fecal samples, but at different relative abundance in infants from both groups (Table 2).

**TABLE 2:** Relative abundance of main bacterial phyla and genera in fecal samples from healthy control and bronchiolitis groups.

	Healthy (*n* = 17)		Bronchiolitis (*n* = 46)		
Bacterial taxon	Prevalence	Relative abundance	Prevalence	Relative abundance	*p* value*
Firmicutes	17 (100)	41.44 (25.72–65.46)	46 (100)	40.4 (30.46–49.37)	0.850
*Streptococcus*	17 (100)	6.08 (1.63–29.13)	46 (100)	8.27 (1.09–15.15)	0.938
*Enterococcus*	17 (100)	0.78 (0.06–3.33)	46 (100)	1.24 (0.19–7.42)	0.210
*Veillonella*	17 (100)	0.39 (0.05–1.67)	46 (100)	0.12 (0.01–1.25)	0.072
*Staphylococcus*	17 (100)	**0.30 (0.04–5.79)**	46 (100)	**0.05 (0.04–0.09)**	**0.046**
*Clostridium*	17 (100)	0.25 (0.05–0.55)	46 (100)	0.31 (0.10–0.59)	0.588
*Blautia*	17 (100)	0.06 (0.02–3.99)	46 (100)	0.68 (0.02–3.92)	0.536
*Lactobacillus*	17 (100)	0.05 (0.02–0.54)	46 (100)	0.04 (0.03–1.62)	0.914
*Erysipleothrix*	17 (100)	0.02 (0.01–0.03)	46 (100)	2.42 (0.01–2.20)	0.125
*Ruminococcus*	15 (88)	0.01 (< 0.01–1.31)	46 (100)	0.21 (< 0.01–3.23)	0.183
Proteobacteria	17 (100)	20.8 (6.72–24.94)	46 (100)	14.51 (5.00–34.16)	0.810
*Escherichia*	17 (100)	2.94 (0.01–7.90)	46 (100)	1.90 (0.10–9.42)	0.853
*Serratia*	17 (100)	0.55 (0.11–1.79)	46 (100)	0.50 (0.07–1.40)	0.951
*Enterobacter*	17 (100)	0.30 (0.14–3.65)	46 (100)	0.33 (0.07–2.79)	0.699
*Klebsiella*	17 (100)	0.09 (0.01–0.66)	46 (100)	0.06 (0.01–1.71)	0.951
*Haemophilus*	17 (100)	**0.04 (0.03–0.08)**	46 (100)	**0.03 (0.03–0.04)**	**0.031**
*Mannheimia*	15 (88)	< 0.01 (<0.01–<0.01)	44 (96)	< 0.01 (<0.01–<0.01)	0.770
Actinobacteria	17 (100)	19.78 (5.88–36.40)	46 (100)	27.55 (10.07–47.96)	0.470
*Bifidobacterium*	17 (100)	18.09 (5.43–34.70)	46 (100)	26.59 (9.37–46.26)	0.386
*Corynebacterium*	17 (100)	0.02 (0.02–0.04)	46 (100)	0.02 (0.02–0.04)	0.380
*Collinsella*	16 (94)	< 0.01 (<0.01–1.88)	46 (100)	0.01 (< 0.01–3.58)	0.072
*Eggerthella*	13 (76)	** < 0.01 (<0.01–<0.01)**	43 (93)	**0.27 (< 0.01–1.12)**	**0.006**
Other phyla	17 (100)	0.11 (0.10–0.24)	46 (100)	0.16 (0.12–0.37)	0.160
*Bacteroides*	17 (100)	0.01 (0.01–6.96)	46 (100)	0.02 (0.01–2.15)	0.853
*Akkermansia*	14 (82)	< 0.01 (<0.01–<0.01)	37 (80)	< 0.01 (<0.01–<0.01)	0.647
Minor genera	17 (100)	9.68 (5.41–17.40)	46 (100)	5.90 (3.40–9.41)	0.107
Unclassified phyla	17 (100)	1.2 (1.07–1.74)	46 (100)	1.27 (1.12–1.83)	0.650
Unclassified genera	17 (100)	6.26 (5.94–8.08)	46 (100)	6.77 (5.60–8.29)	0.988

Bacterial taxon is phylum or genus. The prevalence is expressed as the number (percentage) of samples in which OTUs of the bacterial taxa were detected and the relative abundance of the bacterial taxa as the median and the interquartile range (IQR). *Wilcoxon rank sum tests, with Bonferroni adjustment, to evaluate differences in the relative abundance of phylum or genera. Bold font indicates values having differences that are statistically significant.

In fecal samples, the strongest correlation was registered in the phylum Proteobacteria, and more specifically between the pairs *Escherichia*/*Serratia* (ρ = 0.97). *Haemophilus* and *Mannheimia* were also positively correlated although the correlation coefficient (ρ = 0.52) was lower than in nasal samples (Figure 5). The relative abundances of bacterial genera in nasal and fecal samples showed very low correlation coefficients (Figure 5).

### Associations Among Nasal Bacterial Microbiota and Demographic and Clinical Variables in Infants With Bronchiolitis

**FIGURE 7:**
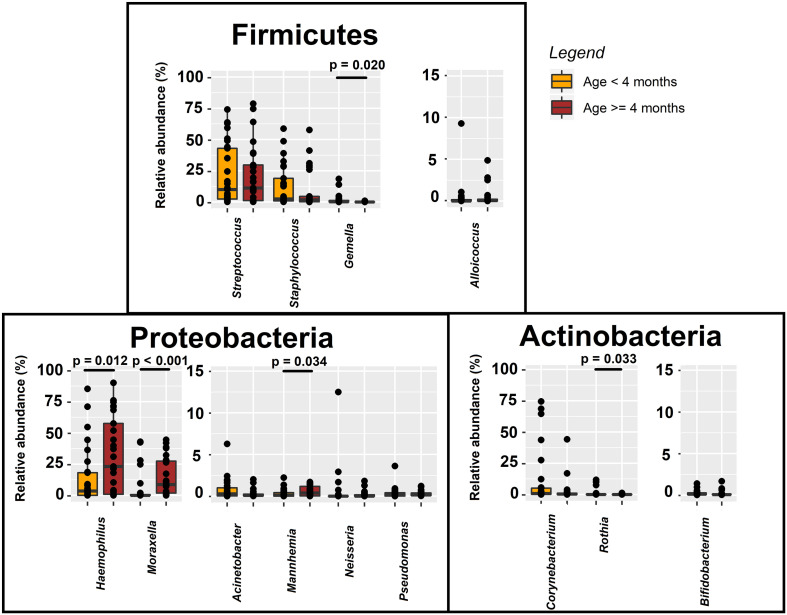
Relative abundance of phylum- and genus-level sequences in nasal samples from bronchiolitis infants aged < 4 years (*n* = 29; orange) and ≥ 4 years (*n* = 29; red). Significant differences between samples from both groups of infants are indicated (Wilcoxon rank sum tests).

The nasal bacterial microbiota was compared between infants aged < 4 and ≥ 4 months because by age 4 months most infants begin eating solid food as a complementary feeding. Differences in the nasal bacterial microbiota profile between infants aged < 4 or ≥ 4 months were found for *Haemophilus* and *Moraxella*, and the minority genera *Gemella*, *Rothia* and *Mannheimia* (Figure 7). The relative abundance of *Haemophilus* in nasal secretions was higher for infants aged ≥ 4 months than for those aged < 4 months (median [IQR] = 23.10% [0.89–57.56%] and 3.47% [0.03–18.13%], respectively; Wilcoxon rank sum tests, *p* = 0.012) (Figure 7) and, also, when the infant had one or more siblings compared to infants who did not have any siblings (18.99% [1.96–44.47%] and 3.19% [0.17–14.18%], respectively; Wilcoxon rank sum tests, *p* = 0.043). The relative content of *Moraxella* was higher in infants attending nursery than in those who did not (median [IQR] = 13.94% [6.16–29.68%] and 0.33% [0.02–9.68%], respectively; Wilcoxon rank sum tests, *p* = 0.013).

Infants requiring hospitalization for > 1 week had about 24-, 9-, and 3-fold relative abundance of *Haemophilus, Mannheimia* and *Corynebacterium* than those requiring < 1 week (Wilcoxon rank sum tests, *p* = 0.013, *p* = 0.045, and *p* = 0.048, respectively) (Table 3). The relative content of *Acinetobacter* was about 3- or 5-fold in infants requiring PICU and/or respiratory support (Wilcoxon rank sum tests, *p* = 0.019 and *p* = 0.028, respectively) and in those diagnosed with pneumonia (Wilcoxon rank sum test, *p* = 0.036). Finally, wheezing was associated to higher abundance of *Moraxella* (median [IQR] = 8.71% (1.82–32.20%) in wheezing infants and 0.35% (0.02–10.59%) in those who did not wheeze; Wilcoxon rank sum test, *p* = 0.038). Wheezing infants also had higher content of *Haemophilus* and *Mannheimia* in nasal samples. On the other hand, no differences in the bacterial microbiota profile were observed in infants with bronchiolitis according to the exposure to tobacco smoke (Table 3).

**TABLE 3:** Bivariate analysis between clinical variables and the relative abundance of the main bacterial genera detected in nasal samples of infants with bronchiolitis.

Phylum Proteobacteria											
		*Haemophilus*		*Moraxella*		*Acinetobacter*		*Mannheimia*		*Pseudomonas*	
Variable	*n* (%)^#^	Median (IQR)	*P*^†^	Median (IQR)	*P*	Median (IQR)	*P*	Median (IQR)	*P*	Median (IQR)	*P*
*Temperature*											
≤37°C	27 (50)	1.70 (0.03–30.90)	0.058	0.36 (0.01–17.32)	0.204	0.13 (0.02–0.45)	0.243	**0.06 (<0.01–0.52)**	**0.039**	0.13 (0.02–0.35)	0.287
>37°C	27 (50)	18.10 (3.02–44.5)		2.0 (0.12–26.4)		0.22 (0.05–0.95)		**0.28 (0.10–0.92)**		0.23 (0.06–0.49)	
*Score*											
≤8	31 (57)	4.15 (0.51–44.51)	0.694	9.12 (0.02–11.70)	0.340	0.09 (0.02–0.45)	0.130	0.18 (0.02–0.81)	0.707	**0.12 (0.02–0.23)**	**0.045**
>8	23 (43)	10.30 (0.44–37.11)		1.95 (0.22–26.40)		0.20 (0.09–0.95)		0.20 (0.04–0.74)		**0.32 (0.08–0.52)**	
*Severity score*											
Moderate	26 (48)	3.53 (0.51–39.70)	0.387	0.60 (0.02–11.70)	0.653	0.08 (0.03–0.45)	0.246	0.11 (0.02–0.72)	0.377	0.12 (0.02–0.23)	0.123
Severe	28 (52)	18.12 (0.62–40.80)		0.92 (0.13–25.60)		0.20 (0.08–0.94)		0.26 (0.05–0.82)		0.31 (0.07–0.43)	
*Hospitalization stay*											
<7 days	26 (48)	**0.83 (0.16–18.00)**	**0.013**	1.34 (0.01–17.33)	0.959	0.13 (0.04–0.26)	0.246	**0.04 (0.01–0.38)**	**0.045**	0.21 (0.08–0.50)	0.416
≥7 days	28 (52)	**20.01 (3.33–48.20)**		0.59 (0.29–16.80)		0.28 (0.03–1.11)		**0.35 (0.10–0.91)**		0.13 (0.03–0.37)	
*PICU admission*											
No	43 (80)	4.36 (0.51–44.54)	0.872	0.59 (0.02–11.72)	0.555	**0.12 (0.02–0.45)**	**0.019**	0.18 (0.03–0.91)	0.957	0.13 (0.02–0.34)	0.088
Yes	11 (20)	10.32 (0.44–36.30)		1.95 (0.16–27.90)		**0.42 (0.17–1.23)**		0.20 (0.06–0.52)		0.39 (0.09–0.66)	
*Respiratory support*											
No	44 (81)	3.41 (0.59–42.13)	0.929	0.59 (0.03–11.14)	0.449	**0.13 (0.03–0.44)**	**0.028**	0.19 (0.03–0.86)	0.722	**0.10 (0.03–0.33)**	**0.043**
Yes	10 (19)	9.54 (0.44–27.10)		5.33 (0.16–27.89)		**0.57 (0.17–1.23)**		0.32 (0.06–0.42)		**0.25 (0.29–0.66)**	
*Pneumonia*											
No	39 (72)	2.73 (0.19–44.52)	0.706	1.24 (0.02–11.72)	0.401	**0.09 (0.02–0.36)**	**0.036**	0.18 (0.02–0.92)	0.721	0.13 (0.03–0.35)	0.329
Yes	15 (28)	10.31 (2.73–27.10)		0.59 (0.04–38.50)		**0.42 (0.14–1.00)**		0.24 (0.10–0.56)		0.23 (0.06–0.47)	
*Recurrent wheezing*											
No	43 (80)	3.87 (0.19–36.32)	0.052	**0.35 (0.02–10.59)**	**0.038**	0.17 (0.03–0.88)	0.485	0.14 (0.02–0.72)	0.076	0.14 (0.02–0.41)	0.555
Yes	11 (20)	21.3 (8.76–68.31)		**8.71 (1.82–32.20)**		0.12 (0.02–0.56)		0.56 (0.20–0.92)		0.32 (0.08–0.46)	
Tobacco exposure*											
No	36 (67)	4.01 (0.38–27.25)	0.219	0.24 (0.01–9.44)	0.108	0.17 (0.04–0.96)	0.217	0.13 (0.03–0.60)	0.288	0.17 (0.02–0.42)	0.670
Yes	16 (30)	22.56 (2.73–40.8)		5.75 (0.51–26.47)		0.13 (0.02–0.24)		0.33 (0.11–0.79)		0.18 (0.06–0.35)	

Phylum Actinobacteria											
		*Corynebacterium*		*Rothia*		*Bifidobacterium*	
Variable	n (%)^#^	Median (IQR)	*P*	Median (IQR)	*P*	Median (IQR)	*P*
*Temperature*							
≤37°C	27 (50)	0.10 (0.02–5.07)	0.328	0.07 (<0.01–0.44)	0.070	0.10 (0.07–0.20)	0.462
>37°C	27 (50)	0.73 (0.27–2.26)		<0.01 (0.00–0.08)		0.10 (0.08–0.38)	
*Score*							
≤8	31 (57)	**0.38 (0.03–1.02)**	**0.049**	<0.01 (<0.01–0.31)	0.254	0.10 (0.07–0.22)	0.720
>8	23 (43)	**1.08 (0.26–12.32)**		<0.01 (0.00–0.10)		0.10 (0.08–0.29)	
*Severity score*							
Moderate	26 (48)	**0.27 (0.02–0.84)**	**0.018**	0.08 (<0.01–0.44)	0.072	0.12 (0.07–0.18)	0.416
Severe	28 (52)	**0.94 (0.29–5.33)**		<0.01 (0.00–0.08)		0.10 (0.08–0.33)	
*Hospitalization stay*							
<7 days	26 (48)	**0.34 (0.02–0.84)**	**0.048**	<0.01 (0.00–0.44)	0.649	0.14 (0.07–0.22)	0.931
≥7 days	28 (52)	**0.93 (0.27–3.16)**		0.02 (0.00–0.10)		0.10 (0.08–0.24)	
*PICU admission*							
No	43 (80)	0.41 (0.15–2.04)	0.540	<0.01 (0.00–0.14)	0.494	0.12 (0.07–0.23)	0.675
Yes	11 (20)	0.69 (0.03–5.07)		0.04 (<0.01–0.10)		0.09 (0.08–0.13)	
*Respiratory support*							
No	44 (81)	0.41 (0.12–1.85)	0.306	<0.01 (0.00–0.18)	0.839	0.12 (0.07–0.22)	0.593
Yes	10 (19)	1.47 (0.39–5.07)		0.03 (<0.01–0.10)		0.09 (0.08–0.10)	
*Pneumonia*							
No	39 (72)	0.59 (0.15–3.88)	0.481	**0.06 (<0.01–0.31)**	**0.015**	0.10 (0.07–0.18)	0.186
Yes	15 (28)	0.41 (0.10–1.08)		**<0.01 (0.00–<0.01)**		0.13 (0.07–0.82)	
*Recurrent wheezing*							
No	43 (80)	0.69 (0.03–2.44)	0.660	0.02 (0.00–0.31)	0.283	0.10 (0.08–0.20)	0.644
Yes	11 (20)	0.33 (0.19–1.47)		<0.01 (0.00–0.10)		0.09 (0.07–0.23)	
Tobacco exposure*							
No	36 (67)	0.56 (0.21–0.16)	0.457	0.01 (<0.01–0.16)	0.288	0.10 (0.07–0.20)	0.597
Yes	16 (30)	0.35 (0.03–1.61)		<0.01 (<0.01–0.08)		0.12 (0.08–0.21)	

PICU, pediatric intensive care unit. Values of the relative abundance of the OTUs assigned to the bacterial genus are median (IQR). ^#^n (%), number (percentage) of participants included in each category. ^†^Wilcoxon rank sum tests to assess the association between each bacterial genus and demographic and clinical variables. *Data for two participants were not available. Bold fonts indicate values having statistically significant differences.

### Bronchiolitis-Related Immune Signatures

Overall, the immunological profile of nasal samples from the bronchiolitis group was more complex than in healthy individuals. There were statistically significant differences in the prevalence of BAFF/TNFSF13B IL11, IL22, IL32, LIGHT/TNFSF14, MMP-2, MMP-3, TSLP, TWEAK/TNFSF12, sCD163, sIL6Rα, sTNF-R1, and sTNF-R2 (Chi-squared or Fisher exact tests, *p* > 0.050) (Table 4). In addition, the concentrations of BAFF/TNFSF13B and sTNF-R1 were also higher in the bronchiolitis group (Wilcoxon rank sum tests, *p* < 0.010) (Table 4). IL8 and osteopontin were present in a large number of samples in both groups, but IL-8 levels were about 10-fold higher in the bronchiolitis group while the opposite was noted for osteopontin (Table 4).

**TABLE 4:** Prevalence (%) and concentration of immune factors in nasal secretion samples from healthy control and bronchiolitis groups.

	Healthy (*n* = 10)		Bronchiolitis (*n* = 45)			
Immune factor	n (%)	Median (IQR)	n (%)	Median (IQR)	*p* value#	*p* value‡
APRIL/TNFSF13, μg/L	9 (90)	6.78 (4.31–8.41)	28 (62)	11.06 (3.73–29.05)	0.140	0.203
BAFF/TNFSF13B, μg/L	**7 (70)**	**0.77 (0.35–1.22)**	**45 (100)**	**2.60 (1.41–4.40)**	**0.005**	**0.006**
Chitinase 3-like 1, μg/L	7 (70)	1.02 (0.78–2.88)	40 (89)	3.00 (1.65–4.97)	0.149	0.107
IFNα2, ng/L	1 (10)	26.83	5 (11)	21.79 (20.55–30.47)	1.000	–
IFNβ, ng/L	0 (0)	–	1 (2)	4.86	1.000	–
IFNγ, ng/L	0 (0)	–	1 (2)	1.93	1.000	–
IL2, ng/L	0 (0)	–	1 (2)	0.82	1.000	–
IL8, μg/L	8 (80)	**0.11 (0.03– 0.44)**	42 (93)	**1.04 (0.29–3.70)**	0.220	**0.005**
IL10, ng/L	1 (10)	0.49	14 (31)	0.77 (0.63–1.27)	0.255	–
IL11, ng/L	**0 (0)**	–	**16 (36)**	1.35 (0.35–2.29)	**0.048**	–
IL12p40, ng/L	0 (0)	–	8 (18)	3.34 (2.83–4.61)	0.326	–
IL12p70, ng/L	0 (0)	–	5 (11)	0.13 (0.13–0.14)	0.572	–
IL19, μg/L	7 (70)	0.30 (0.24–0.94)	42 (93)	0.14 (0.07–0.78)	0.066	0.137
IL20, ng/L	0 (0)	–	5 (11)	34.02 (25.58–67.82)	0.572	–
IL22, ng/L	**0 (0)**	–	**17 (38)**	4.94 (4.24–8.04)	**0.022**	–
IL26, ng/L	0 (0)	–	10 (22)	1.91 (1.53–2.70)	0.179	–
IL27p28, ng/L	0 (0)	–	7 (16)	1.26 (1.16–2.40)	0.328	–
IL28A/IFNλ2, ng/L	0 (0)	–	4 (9)	7.27 (3.20–117.48)	0.584	–
IL29/IFNλ1, ng/L	0 (0)	–	12 (27)	4.10 (3.53–6.44)	0.096	–
IL32, ng/L	**1 (10)**	2.38	**33 (73)**	7.27 (5.61–10.17)	**< 0.001**	–
IL34, ng/L	0 (0)	–	6 (13)	33.12 (27.16–41.97)	0.347	–
IL35, μg/L	5 (50)	0.81 (0.42–1.06)	21 (47)	0.01 (0.01–0.6)	1.000	0.348
LIGHT/TNFSF14, ng/L	**1 (10)**	0.71	**29 (64)**	1.21 (0.76–1.98)	**0.003**	–
MMP-1, ng/L	0 (0)	–	11 (24)	18.80 (16.58–27.89)	0.184	–
MMP-2, ng/L	**1 (10)**	47.76	**32 (71)**	131.97 (70.55–341.45)	**< 0.001**	–
MMP-3, ng/L	**0 (0)**	–	**19 (42)**	41.35 (34.92–86.33)	**0.023**	–
Osteocalcin, ng/L	0 (0)	–	2 (4)	14.79 (13.45–16.12)	1.000	–
Osteopontin, μg/L	5 (50)	**0.97 (0.65–2.63)**	36 (80)	**0.14 (0.12–0.16)**	0.101	**0.002**
Pentraxin 3, ng/L	5 (50)	7.26 (3.21–42.54)	27 (60)	23.64 (1.40–218.40)	0.726	0.595
TSLP, ng/L	**0 (0)**	–	**19 (42)**	1.74 (1.19–2.80)	**0.023**	–
TWEAK/TNFSF12, ng/L	**3 (30)**	2.25 (1.55–55.12)	**36 (80**)	5.45 (2.23–12.63)	**0.004**	0.753
gp130/sIL-6Rβ, ng/L	7 (70)	101.76 (65.26–235.98)	42 (93)	335.86 (140.26–725.89)	0.066	0.119
sCD30/TNFRSF8, ng/L	0 (0)	–	10 (22)	1.76 (1.12–6.04)	0.179	–
sCD163, ng/L	**1 (10)**	94.71	**34 (76)**	559.64 (182.61–1662.57)	**< 0.001**	–
sIL-6Rα, ng/L	**4 (40)**	9.65 (2.80–41.38)	**39 (87)**	38.23 (10.05–90.25)	**0.004**	0.300
sTNF-R1, ng/L	7 (70)	**39.51 (12.74–83.92)**	41 (91)	**291.99 (92.99–595.03)**	0.104	**0.003**
sTNF-R2, ng/L	**1 (10)**	5.33	**36 (80)**	148.84 (21.29–288.97)	**< 0.001**	–

*APRIL/TNFSF13, A proliferation-inducing ligand/tumor necrosis factor ligand superfamily member 13; BAFF/TNFSF13B, B-cell activating factor/tumor necrosis factor ligand superfamily member 13B; gp130/sIL-6Rβ, glycoprotein 130/soluble interleukin 6 receptor β; IFN, interferon; IL, interleukin; LIGHT/TNFSF14, tumor necrosis factor superfamily member 14; MMP, matrix metalloproteinase; sCD30/TNFRSF8, soluble cluster of differentiation 30/Tumor Necrosis Factor Receptor Superfamily, Member 8; sCD163, soluble cluster of differentiation 163; sIL-6Rα, soluble interleukin 6 receptor α; sTNF-R1, soluble tumor necrosis factor receptor 1; sTNF-R2, soluble tumor necrosis factor receptor 2; TSLP, thymic stromal lymphopoietin; TWEAK/TNFSF12, tumor necrosis factor superfamily member 12. The prevalence is expressed as the number (percentage) of samples in which the immunological compound was detected and the relative abundance of the immunological compound as the median and the interquartile range (IQR). ^#^Chi-squared or Fisher exact tests were used to evaluate differences in prevalence of the analyzed parameters. **^‡^**Wilcoxon rank sum tests were used to evaluate differences in concentration of the analyzed parameters. Bold font indicates values having differences that are statistically significant.*

The frequency of detection of most immune factors was higher in fecal samples from healthy individuals than in the bronchiolitis group, opposite to nasal samples. BAFF/TNFSF13B was detected in almost all fecal samples but the concentration in the bronchiolitis group was approximately double that in healthy infants, similarly to nasal samples (Table 5).

**TABLE 5:** Prevalence (%) and concentration of immune factors in fecal samples from healthy control and bronchiolitis groups.

	Healthy (*n* = 15)		Bronchiolitis (*n* = 48)			
Immune factor	n (%)	Median (IQR)	n (%)	Median (IQR)	*p* value#	*p* value‡
APRIL/TNFSF13, μg/L	**6 (40)**	2.38 (2.00–2.65)	**4 (8)**	1.58 (1.37–2.27)	**0.008**	0.336
BAFF/TNFSF13B, μg/L	15 (100)	**0.85 (0.50–1.45)**	47 (98)	**1.89 (1.29–2.82)**	1.000	**0.011**
Chitinase 3-like 1, μg/L	15 (100)	1.56 (0.19–3.48)	42 (88)	0.99 (0.42–2.63)	0.321	0.986
IFNα2, ng/L	2 (13)	37.30 (32.61–42.00)	0 (0)	–	0.054	–
IFNβ, ng/L	**8 (53)**	9.34 (5.61–18.83)	**6 (13)**	11.07 (7.62–17.12)	**0.002**	0.796
IFNγ, ng/L	**5 (33)**	16.86 (16.64–19.72)	**2 (4)**	19.13 (15.38–22.87)	**0.007**	0.699
IL2, ng/L	2 (13)	15.17 (12.01–18.34)	0 (0)	–	0.054	–
IL8, ng/L	4 (27)	23.15 (16.20–30.35)	8 (17)	21.82 (14.26–98.67)	0.457	0.734
IL10, ng/L	3 (20)	5.31 (4.53–8.30)	2 (4)	4.30 (4.04–4.56)	0.083	0.564
IL11, ng/L	3 (20)	0.53 (0.50–2.14)	4 (8)	0.59 (0.51–0.72)	0.342	1.000
IL12p40, ng/L	5 (33)	32.84 (22.69–69.40)	7 (15)	26.12 (22.69–35.13)	0.137	0.465
IL12p70, ng/L	3 (20)	2.32 (2.15–2.79)	2 (4)	2.14 (1.65–2.62)	0.083	0.564
IL19, ng/L	**3 (20)**	17.84 (15.66–79.82)	**0 (0)**	–	**0.011**	–
IL20, ng/L	**11 (73)**	17.84 (12.52–21.19)	**19 (40)**	17.35 (11.31–21.32)	**0.037**	0.880
IL22, ng/L	1 (7)	22.1	0 (0)	–	0.238	–
IL26, ng/L	2 (13)	30.95 (28.67–33.22)	2 (4)	26.21 (25.92–26.49)	0.238	0.439
IL27p28, ng/L	2 (13)	55.46 (34.11–76.80)	9 (19)	18.93 (11.24–35.20)	0.721	0.480
IL28A/IFNλ2, ng/L	2 (13)	13.77 (11.11–16.42)	3 (6)	9.81 (9.75–11.15)	0.585	1.000
IL29/IFNλ1, ng/L	**5 (33)**	50.46 (28.18–62.00)	**5 (10)**	28.18 (27.16–36.82)	**0.049**	0.530
IL32, ng/L	**11 (73)**	17.34 (14.97–21.42)	**16 (33)**	25.35 (11.38–32.74)	**0.008**	0.587
IL34, ng/L	1 (7)	753.61	9 (19)	777.19 (750.98–944.30)	0.428	–
IL35, ng/L	3 (20)	**222.05 (220.33–251.99)**	3 (6)	**89.72 (78.15–133.29)**	0.141	**0.050**
LIGHT/TNFSF14, ng/L	**11 (73)**	63.18 (37.46–165.69)	**11 (23)**	54.59 (44.60–75.11)	**0.001**	0.533
MMP-1, μg/L	2 (13)	1.97 (1.80– 2.13)	1 (2)	1.19	0.138	–
MMP-2, μg/L	6 (40)	0.77 (0.54–0.89)	7 (15)	0.71 (0.54–0.93)	0.062	0.943
MMP-3, μg/L	**5 (33)**	0.55 (0.26–0.64)	**5 (10)**	0.32 (0.22–0.33)	**0.049**	0.347
Osteocalcin, μg/L	**4 (27)**	0.18 (0.14–0.23)	**2 (4)**	0.13 (0.10–0.16)	**0.025**	0.355
Osteopontin, μg/L	6 (40)	0.71 (0.52–0.92)	7 (15)	0.44 (0.42–0.62)	0.062	0.063
Pentraxin 3, ng/L	**15 (100)**	31.06 (23.32–43.24)	**25 (52)**	28.35 (15.32–46.39)	**0.001**	0.379
TSLP, ng/L	**7 (47)**	11.68 (9.25–22.10)	**7 (15)**	12.26 (9.00–14.81)	**0.015**	0.749
TWEAK/TNFSF12, μg/L	15 (100)	0.62 (0.22–1.11)	46 (96)	0.29 (0.10–0.77)	1.000	0.227
gp130/sIL-6Rβ, ng/L	5 (33)	93.86 (70.96–99.92)	17 (35)	90.92 (45.88–153.35)	1.000	0.969
sCD30/TNFRSF8, ng/L	4 (27)	10.54 (7.07–16.77)	5 (10)	7.67 (7.47–7.86)	0.198	1.000
sCD163, μg/L	6 (40)	0.94 (0.60–1.63)	13 (27)	0.87 (0.70–1.77)	0.520	0.930
sIL-6Rα, ng/L	4 (27)	14.38 (13.74–17.26)	5 (10)	10.88 (10.51–15.29)	0.198	0.462
sTNF-R1, ng/L	8 (53)	59.02 (34.09–137.82)	30 (63)	110.39 (59.70–369.67)	0.558	0.100
sTNF-R2, ng/L	2 (13)	56.25 (49.19–63.31)	6 (13)	101.21 (36.27–280.36)	1.000	1.000

*APRIL/TNFSF13, A proliferation-inducing ligand/tumor necrosis factor ligand superfamily member 13; BAFF/TNFSF13B, B-cell activating factor/tumor necrosis factor ligand superfamily member 13B; gp130/sIL-6Rβ, glycoprotein 130/soluble interleukin 6 receptor β; IFN, interferon; IL, interleukin; LIGHT/TNFSF14, tumor necrosis factor superfamily member 14; MMP, matrix metalloproteinase; sCD30/TNFRSF8, soluble cluster of differentiation 30/Tumor Necrosis Factor Receptor Superfamily, Member 8; sCD163, soluble cluster of differentiation 163; sIL-6Rα, soluble interleukin 6 receptor α; sTNF-R1, soluble tumor necrosis factor receptor 1; sTNF-R2, soluble tumor necrosis factor receptor 2; TSLP, thymic stromal lymphopoietin; TWEAK/TNFSF12, tumor necrosis factor superfamily member 12. The prevalence is expressed as the number (percentage) of samples in which the immunological compound was detected and the relative abundance of the immunological compound as the median and the interquartile range (IQR). ^#^Chi-squared or Fisher exact tests were used to evaluate differences in prevalence of the analyzed parameters. **^‡^**Wilcoxon rank sum tests were used to evaluate differences in concentration of the analyzed parameters. Bold font indicates values having differences that are statistically significant.*

## Discussion

Although bronchiolitis is a viral infection, emerging evidence indicates that the microbiota may be associated to its pathogenesis. In this study, the composition of the bacterial microbiota and the immune profiles of nasal and fecal samples from infants with a RSV-associated bronchiolitis were assessed and compared with those from healthy ones.

All the nasal samples that were characterized by the dominance of *Haemophilus* or by the abundance (> 20%) of *Moraxella* sequences had been obtained from the bronchiolitis group. In previous studies, bronchiolitis-suffering infants with a *Haemophilus*- or a *Moraxella*-dominant profile had significantly higher rates of incidence and severity of acute respiratory infections, intensive care use or prolonged hospital stay, in comparison with the profiles of bacterial microbiota dominated by other bacteria (Hasegawa et al., 2016a; Teo et al., 2015). Our results confirm that an *Haemophilus*-dominant profile is a common feature of RSV-associated bronchiolitis (Hasegawa et al., 2016a; McGillivary et al., 2009; Vissing et al., 2013).

In contrast with other authors, we did not find an association between RSV-associated bronchiolitis and an abundance of *Streptococcus* sequences (Teo et al., 2015; de Steenhuijsen Piters et al., 2016). In addition, the abundance of *Mannheimia* sequences was significantly higher in the bronchiolitis group. Although this genus contains species involved in respiratory infections in a wide spectrum of mammals, such as *M.* (formerly *Pasteurella*) *haemolytica*, this is the first work describing an enrichment in *Mannheimia* sequences among infants with RSV-associated bronchiolitis. This finding must be confirmed in future studies. In the bronchiolitis group, relative abundances of *Staphylococcus* and *Corynebacterium* were significantly lower than in samples from the control group. A lower nasal abundance of *Corynebacterium* and *Staphylococcus* among infants with bronchiolitis has already been described (Hasegawa et al., 2016a; Biesbroek et al., 2014; Bosch et al., 2017). A recent study has shown that the degree of alteration of both the structure and networks of the nasal microbiota was associated to the severity of the RSV-associated bronchiolitis disease (Schippa et al., 2020).

In contrast to the nasal microbiota, the fecal microbiota of infants with bronchiolitis was similar to that of healthy infants. This finding may be limited by the sample size, especially in relation to the healthy controls. The nasal cavity drains to the pharynx, which is connected to both the trachea and the esophagus. Following the later route, nasopharyngeal bacteria may reach the gut (Proctor and Relman, 2017). However, while there are high levels of similarity between microbial communities living in the oral cavity, stomach and the gut, such similarities are not found between nasal and gut communities (Bik et al., 2006; Ding and Schloss, 2014; Bassis et al., 2015). However, Hasegawa et al. (2016b) identified 4 distinct profiles (*Escherichia*-dominant, *Bifidobacterium*-dominant, *Enterobacter*/*Veillonella*-dominant and *Bacteroides*-dominant) when the microbial profiles of bronchiolitis patients where compared to those of healthy controls. Interestingly, the proportion of bronchiolitis was highest among infants with the *Bacteroides*-dominant profile while it was lowest in those with the *Enterobacter*/*Veillonella*-dominant profile. More studies are required to elucidate or confirm potential relationships between the fecal bacteriome and bronchiolitis.

In relation to the immunological profiles, the concentration of BAFF/TNFSF13B, IL-8, and sTNF.R1 were significant higher in nasal samples from the bronchiolitis group. RSV infection upregulates expression of BAFF/TNFSF13B (Reed et al., 2009), leading to an increase in its concentration in the bronchoalveolar lavage fluid from RSV-infected infants (McNamara et al., 2013). This finding seems to be a consistent feature of the upper and lower respiratory tract responses to infections in infants, and reveals the importance of the airway epithelia in influencing immune and inflammatory responses.

In this study, BAFF was the only immune parameter which concentration was significantly higher among fecal samples from the bronchiolitis group than those from the healthy group. Airways epithelial cells and gut dendritic cells seem to play similar roles in producing BAFF in the respective mucosal surfaces, where it may support IgA production by B cells (Cerutti et al., 2011). Fecal BAFF increases when the gut is inflamed and it has been proposed as a biomarker for monitoring children with inflammatory bowel diseases (Fodor et al., 2020). The results of our study suggest that such an increase may also reflect an inflammatory state in other mucosal surfaces, such as that of the respiratory tract.

In relation to the remaining immune factors, increased IL8 and sTNF.R1 levels, and decreased osteopontin levels, have also been reported in nasal samples from infants with RSV bronchiolitis (Noah et al., 2002; Sampayo-Escobar et al., 2018; Breindahl et al., 2012). However, the number of studies addressing such immune compounds in the RSV context is still low and more studies are required to confirm their relevance as RSV biomarkers.

The main limitation of this study is the limited number of participants according to the high variability of some bacterial microbiota and immunological parameters associated to RSV bronchiolitis. In addition, all participants were recruited from a single center. However, this allowed us to determine the microbiota composition and immunoprofiling (including a very large number of immune compounds) in nasal and fecal samples of the same participants. Despite our efforts, there were missing samples and data, but this fact did not interfere with the performed analyses and the number of participants included in each comparison is adequately indicated in each table and figure. It must be taken into account that the bacteriomes of the upper respiratory and intestinal tracts are dynamic and seem to evolve rapidly during early life (Bosch et al., 2017; Durack and Christophersen, 2020); this fact may exert an influence, which is currently unknown, when results obtained from infants of different ages are compared. In conclusion, the results of this study confirm that RSV-associated bronchiolitis is correlated with an abundance of *Haemophilus* sequences and to an increase in the concentrations of BAFF/TNFSF13B and IL8 in nasal samples. In addition, it suggests the existence of other potential biomarkers, including an increase in *Mannheimia* sequences and in the concentrations of fecal BAFF/TNFSF13B and nasal sTNF.R1 and a decrease in nasal osteopontin. Such markers may be applied in the future to evaluate the response of infants with RSV-bronchiolitis to treatments.

## Data Availability Statement

The raw data supporting the conclusions of this article will be made available by the authors, without undue reservation.

## Ethics Statement

The studies involving human participants were reviewed and approved by Ethics Committee of the Hospital Gregorio Marañón. Written informed consent to participate in this study was provided by the participants’ legal guardian/next of kin.

## Author Contributions

JR, RR-F, and LF conceived and designed the study, supervised the analyses of the data, drafted the initial manuscript, and reviewed and revised the manuscript. FG-M, MG-S, JP-M, and BT recruited the infants, collected the samples, the demographic and clinical data, and reviewed and revised the manuscript. CA and MA performed the metataxonomic and immunological analysis, including initial statistical and bioinformatic analysis, and reviewed and revised the manuscript. All authors discussed the results, commented on the manuscript and approved the submitted version of the manuscript.

## Conflict of Interest

The authors declare that the research was conducted in the absence of any commercial or financial relationships that could be construed as a potential conflict of interest.

## Footnotes

1 https://www.R-project.org
