# Supplementary material for: Nasal and Fecal Microbiota and Immunoprofiling of Infants With and Without RSV Bronchiolitis
Source: Front Microbiol. 2021 Jun 1;12:667832. doi: 10.3389/fmicb.2021.667832 (PMC8203809; doi:10.3389/fmicb.2021.667832)
Supplement: Supplementary file 1 [file Table_1.DOCX]

Supplementary Material

**Nasal and fecal microbiota and immunoprofiling of infants with and without RSV bronchiolitis**

Claudio Alba^1†^, Marina Aparicio^1†^, Felipe González-Martínez^2,3^, María Isabel González-Sánchez^2,3^, Jimena Pérez-Moreno^2,3^, Blanca Toledo del Castillo^2,3^, Juan Miguel Rodríguez^1^, Rosa Rodríguez-Fernández^2,3^, Leonides Fernández^4*^

**Supplementary Table 1.** Relative abundance of main bacterial phyla and genera in nasal secretion samples from healthy control and bronchiolitis groups.

|  | **Healthy (n = 14)** | | | **Bronchiolitis (n = 54)** | | |  |
| --- | --- | --- | --- | --- | --- | --- | --- |
| **Bacterial taxon** | **Prevalence** | **Relative abundance** | **Prevalence** | | **Relative abundance** | ***p* value^*^** | |
| Firmicutes | 14 (100) | **61.02 (49.84-74.03)** | 54 (100) | | **37.41 (12.09-58.69)** | **0.010** | |
| *Staphylococcus* | 14 (100) | **21.94 (1.85-38.56)** | 54 (100) | | **2.19 (0.52-17.92)** | **0.023** | |
| *Streptococcus* | 14 (100) | 7.56 (2.55-32.59) | 54 (100) | | 10.14 (1.97-37.81) | 0.649 | |
| *Alloiococcus* | 11 (79) | 0.02 (0.01-14.62) | 43 (80) | | 0.48 (0.011-9.31) | 0.290 | |
| *Alkalibacterium* | 14 (100) | 2.17 (0.07-9.20) | 54 (100) | | 0. 20 (0.06-2.92) | 0.168 | |
| *Gemella* | 14 (100) | 1.17 (0.18-6.97) | 54 (100) | | 0.04 (0.01-0.52) | 0.214 | |
| Actinobacteria | 14 (100) | **9.97 (6.98-36.62)** | 54 (100) | | **4.22 (1.61-10.96)** | **0.019** | |
| *Corynebacterium* | 14 (100) | **4.97 (1.27-32.82)** | 54 (100) | | **0.48 (0.15-2.22)** | **0.007** | |
| *Bifidobacterium* | 14 (100) | **0.16 (0.11-0.28)** | 54 (100) | | **0.10 (0.07-0.21)** | **0.038** | |
| Proteobacteria | 14 (100) | **3.72 (2.48-16.42)** | 54 (100) | | **32.64 (11.21-66.89)** | **<0.001** | |
| *Moraxella* | 14 (100) | 0.10 (0.01-0.59) | 54 (100) | | 0.73 (0.02-15.88) | 0.101 | |
| *Haemophilus* | 14 (100) | **0.23 (0.04-1.36)** | 54 (100) | | **6.77 (0.55-39.06)** | **0.004** | |
| *Mannheimia* | 14 (100) | **0.02 (0.01-0.07)** | 51 (94) | | **0.19 (0.03-0.74)** | **0.011** | |
| Cyanobacteria | 14 (100) | 0.14 (0.09-0.46) | 54 (100) | | 0.10 (0.02-1.30) | 0.485 | |
| Minor phyla | 14 (100) | 0.36 (0.17-1.92) | 54 (100) | | 0.45 (0.20-2.16) | 0.467 | |
| Minor_genera | 14 (100) | 6.36 (4.42-10.63) | 54 (100) | | 9.02 (5.30-22.18) | 1.000 | |
| Unclassified phyla | 14 (100) | **1.58 (1.33-2.35)** | 54 (100) | | **3.39 (1.78-6.69)** | **0.004** | |
| Unclassified genera | 14 (100) | **7.24 (5.75-8.09)** | 54 (100) | | **8.71 (7.05-13.05)** | **0.027** | |

Bacterial taxon is phylum or genus.

The prevalence is expressed as the number (percentage) of samples in which OTUs of the bacterial taxa were detected and the relative abundance as the median and the interquartile range (IQR).

*Wilcoxon rank sum tests, with Bonferroni adjustment, to evaluate differences in the relative abundance of phylum or genera.
